# Association of the *MMP-9* polymorphism and ischemic stroke risk in southern Chinese Han population

**DOI:** 10.1186/s12883-019-1285-7

**Published:** 2019-04-16

**Authors:** Ning Gao, Tie Guo, Han Luo, Guolong Tu, Fanglin Niu, Mengdan Yan, Ying Xia

**Affiliations:** 1 Department of Neurosurgery, Affiliated Haikou Hospital of Xiangya Medical College in Central South University, Haikou, Hainan 570208 China; 2 The First Affiliated Hospital of Zhengzhou University, Zhengzhou, 450052 Henan China; 3 Key Laboratory of Resource Biology and Biotechnology in Western China, Northwest University, Ministry of Education, Xi’an, Shaanxi China

**Keywords:** Ischemic stroke, *MMP-9*, Case-control study, Single nucleotide polymorphism

## Abstract

**Background:**

Stroke is a serious cardiovascular disease and is also the leading cause of long-term disability in developing and developed countries. Because matrix metalloproteinase-9 (*MMP-9*) is associated with the risk of many cardiovascular diseases, we investigated the relationship between single nucleotide polymorphisms (SNPs) in *MMP-9* and the risk of Ischemic stroke (IS) in a southern Chinese Han population.

**Methods:**

This study included 250 stroke patients and 250 healthy controls. Genotyping was performed using the Agena MassARRAY system, and chi-squared tests and genetic models were used to evaluate the associations between *MMP-9* SNPs and the risk of IS. Odds ratio (OR) and 95% confidence intervals (CIs) were calculated by unconditional logistic regression adjusted for age.

**Results:**

Polymorphism rs3787268 was associated with increased the risk of IS. Specifically, the genotype “G/A” significantly correlated with IS risk in the co-dominant model [odds ratio (OR) = 1.62; 95% confidence interval (CI) = 1.10–2.41; *p* = 0.035)], while genotypes “G/A” and “A/A” may increase the risk of IS based on the dominant model (OR = 1.62; 95% CI = 1.12–2.35; *p* = 0.0097). This SNP was also significantly associated with IS risk in the log-additive model (OR = 1.33; 95% CI = 1.03–1.70; *p* = 0.026). Conversely, haplotype “C/G” appears to reduce the risk of IS (OR = 0.71; 95% CI = 0.54–0.95; *p* = 0.019).

**Conclusions:**

Our study showed that the rs3787268 locus in the *MMP-9* gene may increase risk of IS in a southern Chinese Han population and thus provide insight into the IS pathogenesis.

## Background

Stroke is a serious cardiovascular disease with an estimated global mortality of 4.7 million per year [1], and is also the leading cause of long-term disability in developing and developed countries [2]. It occurs when the brain tissue does not get enough oxygen and nutrients [3]. Ischemic stroke (IS) is the most common type of stroke, accounting for about 72–86% of cases [4]. The specific pathogenesis of IS remains unclear, but increasing evidence indicates that both environmental and genetic factors play a crucial role in its etiology. Observational studies have shown that hypertension [5], diabetes [6], smoking [7], drinking [8], hypercholesterolemia [9], lack of exercise among young people [10] may be clinically relevant risk factors for IS. Nevertheless, a large body of scientific research have indicated that IS was greatly affected by genetic factors [11], and gene polymorphisms may regulate the pathophysiological process of IS and confer a small to moderate risk [12].

Matrix metalloproteinases (MMPs) are a family of zinc- and calcium-dependent enzymes with proteolytic activity and can be adjusted by tissue inhibitors [13]. *MMP-9* plays an important part in various cardiovascular diseases [14] and can degrade components of the extracellular matrix, leading to weakening of the fibrous cap [15] and development of cardiovascular and cerebrovascular diseases, including IS, atherosclerosis [16], and neuroinflammation [17]. The research of Zhong et al. showed that certain *MMP-9* polymorphisms may increase the risk of IS in western Guangdong Province, China [18], although Buraczynska et al. did not observe associations between *MMP-9* polymorphisms and IS in a Polish population [19]. However, it is unknown if *MMP-9* polymorphisms are significantly related to IS risk in a southern Chinese Han population.

Therefore, the purpose of this study is to determine if susceptible single nucleotide polymorphisms (SNPs) in the *MMP9* gene are associated with increased risk of IS in a southern Chinese Han population. These results may contribute to further clarify their potential role in IS and provide basis for the early prevention and targeted treatment of IS in a southern Chinese Han population.

## Methods

### Study participants

Our study consisted of 250 stroke patients, who were consecutively recruited between January 2015 and January 2018 at the Haikou Hospital affiliated to Xiangya Medical College of Central South University, with no age or gender restrictions. Controls identified through the annual health assessment were recruited from the same hospital physical examination center between January 2015 and October 2017. They are healthy individuals had no history of cerebrovascular disease or MI, tumor, hypertension, diabetes, etc. All study participants were Han Chinese individuals living in the Hainan province and provided written informed consent. The Ethic Committee of the Haikou Hospital affiliated to Xiangya Medical College of Central South University approved the use of human blood samples for this study.

According to the World Health Organization’s diagnostic criteria, all participants’ IS was confirmed by at least two independent neurologists using computed tomography (CT) scans and/or magnetic resonance imaging (MRI) along with standardized blood tests. Patients with IS were excluded from the study if they had history of transient ischemic attack, coronary artery disease, autoimmune disease, systemic inflammatory disease, malignant tumor and.

### Single nucleotide polymorphisms (SNPs) selection and genotyping

The professional technicians professional technicians collected about 5 mL of peripheral blood samples of each participant into the test tube containing ethylenediamine tetraacetic acid (EDTA) for anticoagulation in a freezer, at − 80 °C.Subsequently, genomic DNA was then extracted from blood samples using the Gold-Mag nanoparticles method (Gold Mag Co. Ltd., Xi’an City, China) according to the manufacturer’s instructions, and the DNA concentration and purity were measured by NanoDrop 2000C (Thermo Scientifc, Waltham, Massachusetts, USA), and finally stored at − 80 °C until analysis. Blood was taken within 5 h after the patient was initial diagnosed with a IS.

In our study, four SNPs rs3787268, rs3918249, rs2274755 and rs3918254 in *MMP-9* were selected for genotyping. Based on the research, it is found that rs3787268 has no correlation with IS in the Polish population [19], but it will increase the risk of IS in the western Guangdong region [18], indicating that the correlation between this site and IS was different in different populations, and it is still different. It is unclear whether the *MMP-9* polymorphism is significantly associated with the IS risk of the southern Chinese Han population, so it was chosen. The choice of rs3918249, rs2274755, and rs3918254 based on the fact that their effects on IS has not been studied. Data management and analysis were performed with Agena MassARRAY Assay Design software v4.0 [10, 11].

### Statistical analyses

All statistical analyses were performed using SPSS v19.0 (IBM Analytics, Chicago, IL, USA) and Microsoft Excel. Allele and genotype frequencies were obtained by direct counts and analyzed with Chi-squared and Fisher’s exact tests. Hardy-Weinberg equilibrium (HWE) for each SNP was determined using an exact test to compare the expected frequencies of genotypes in controls. Association between *MMP-9* polymorphisms and the risk of IS were estimated by computing odds ratios (OR) and 95% confidence intervals (CIs) with unconditional logistic regression analysis. Four models (co-dominant, dominant, recessive, and log-additive) were used to assess these relationships [12]. Linkage disequilibrium (LD) analysis was performed using genotype data from IS patients and controls. All *p* values were two- sided, and *p* < 0.05 indicated statistical significance [13].

## Results

Our study included 250 IS cases (167 males, 83 females; median age at diagnosis: 64.13 years) and 250 controls (152 males, 98 females; median age 48.31 years). As shown in Table 1, we detected no significant difference in the distribution of age or gender between cases and healthy controls (*p* >  0.001).

**Table 1:** Characteristics of patients with ischemic stroke and the control individuals

Variable(s)	Case (*n* = 250)	Control (*n* = 250)	*p* value
Sex N (%)			> 0.001^a^
Male	167	152	
Female	83	98	
Age, year (mean ± SD)	64.13 ± 10.82	48.31 ± 13.31	> 0.001^b^

^a^Two-sided Chi-squared test

^b^Independent samples *t* test

Table 2 summarizes the allele frequencies of tested SNPs among individuals in both case and control groups. All SNP call rates exceeded 98.5%, which were high enough to perform association analyses. In addition to SNP rs2274755 locus (*p-*HWE >  0.05), all tested loci followed HWE at the 5% level. Chi-squared tests indicated rs3787268 was significantly associated with increased IS risk (OR = 1.34, 95% CI = 1.04–1.73; *p* = 0.022).

**Table 2:** Allele frequencies in cases and controls and odds ratio estimates for IS risk

SNP	Gene(s)	Band	Alleles A/B	MAF		HWE ^a^ *p* value	OR (95% CI)	^b^ *p* value
				Case	Control			
rs3918249	*MMP9*	20q13.12	T/C	0.212	0.251	0.09	0.80 (0.59–1.07)	0.142
rs2274755	*MMP9*	20q13.12	T/G	0.116	0.131	0.011	0.88 (0.60–1.28)	0.500
rs3918254	*MMP9*	20q13.12	T/C	0.221	0.227	0.277	0.97 (0.72–1.30)	0.820
rs3787268	*MMP9*	20q13.12	A/G	0.442	0.371	0.134	1.34 (1.04–1.73)	0.022^*^

SNP: single nucleotide polymorphism, Alleles A/B: Minor/Major alleles, MAF: minor allele frequency, OR: odds ratio, CI: confidence interval, HWE: Hardy–Weinberg equilibrium

^*^ *p* ≤ 0.05 indicates statistical significance

^a^ *p* was calculated by exact test

^b^ *p* was calculated by Pearson Chi-squared test

We used four genetic models to analyze the association between the tested SNPs and risk of IS (Table 3). In the co-dominant model, genotype “G/A” of rs3787268 increased the risk of IS 1.62-fold (OR = 1.62; 95% CI = 1.10–2.41; *p* = 0.035). In the dominant model, genotype “G/A - A/A” was associated with a 1.62-fold increase in IS risk (OR = 1.62; 95% CI = 1.12–2.35; *p* = 0.010). The log-additive model indicated rs3787268 increased the risk of IS 1.33-fold (OR = 1.33, 95% CI = 1.03–1.70; *p* = 0.026).

**Table 3:** Relationship between MMP-9 polymorphism and IS risk

SNP	Model	Genotype	control	case	OR (95% CI)	*P*-value	AIC	BIC
rs3787268	Codominant	G/G	104 (41.9%)	77 (30.8%)	1.00	0.035^*^	689.7	702.3
		G/A	104 (41.9%)	125 (50%)	1.62 (1.10–2.41)			
		A/A	40 (16.1%)	48 (19.2%)	1.62 (0.97–2.71)			
	Dominant	G/G	104 (41.9%)	77 (30.8%)	1.00	0.009^*^	687.7	696.1
		G/A-A/A	144 (58.1%)	173 (69.2%)	1.62 (1.12–2.35)			
	Recessive	G/G-G/A	208 (83.9%)	202 (80.8%)	1.00	0.370	693.6	702
		A/A	40 (16.1%)	48 (19.2%)	1.24 (0.78–1.96)			
	Overdominant	G/G-A/A	144 (58.1%)	125 (50%)	1.00	0.071	691.1	699.5
		G/A	104 (41.9%)	125 (50%)	1.38 (0.97–1.97)			
	Log-additive	–	–	–	1.33 (1.03–1.70)	0.026^*^	689.4	697.8
rs3918249	Co-dominant	C/C	145 (58.2%)	155 (62.5%)	1.00	0.240	692.1	704.8
		T/C	83 (33.3%)	81 (32.7%)	0.91 (0.62–1.34)			
		T/T	21 (8.4%)	12 (4.8%)	0.53 (0.25–1.13)			
	Dominant	C/C	145 (58.2%)	155 (62.5%)	1.00	0.330	692	700.5
		T/C-T/T	104 (41.8%)	93 (37.5%)	0.84 (0.58–1.20)			
	Recessive	C/C-T/C	228 (91.6%)	236 (95.2%)	1.00	0.110	690.4	698.8
		T/T	21 (8.4%)	12 (4.8%)	0.55 (0.27–1.15)			
	Over-dominant	C/C-T/T	166 (66.7%)	167 (67.3%)	1.00	0.870	693	701.4
		T/C	83 (33.3%)	81 (32.7%)	0.97 (0.67–1.41)			
	Log-additive	–	–	–	0.81 (0.61–1.08)	0.160	691	699.4
rs2274755	–	G/G	184 (73.9%)	191 (76.7%)	1.00	0.470	693.8	702.3
		G/T	65 (26.1%)	58 (23.3%)	0.86 (0.57–1.29)			
rs3918254	Co-dominant	C/C	152 (61%)	152 (61%)	1.00	0.830	696	708.6
		T/C	81 (32.5%)	84 (33.7%)	1.04 (0.71–1.52)			
		T/T	16 (6.4%)	13 (5.2%)	0.81 (0.38–1.75)			
	Dominant	C/C	152 (61%)	152 (61%)	1.00	NA	694.4	702.8
		T/C-T/T	97 (39%)	97 (39%)	1.00 (0.70–1.43)			
	Recessive	C/C-T/C	233 (93.6%)	236 (94.8%)	1.00	0.570	694	702.5
		T/T	16 (6.4%)	13 (5.2%)	0.80 (0.38–1.70)			
	Over-dominant	C/C-T/T	168 (67.5%)	165 (66.3%)	1.00	0.780	694.3	702.7
		T/C	81 (32.5%)	84 (33.7%)	1.06 (0.73–1.53)			
	Log-additive	–	–	–	0.97 (0.72–1.29)	0.820	694.3	702.7

ORs, odds ratios; CI: confidence interval; AIC: Akaike’s Information criterion; BIC: Bayesian Information criterion

^*^p value ≤0.05 indicates statistical significance

**Fig. 1:**
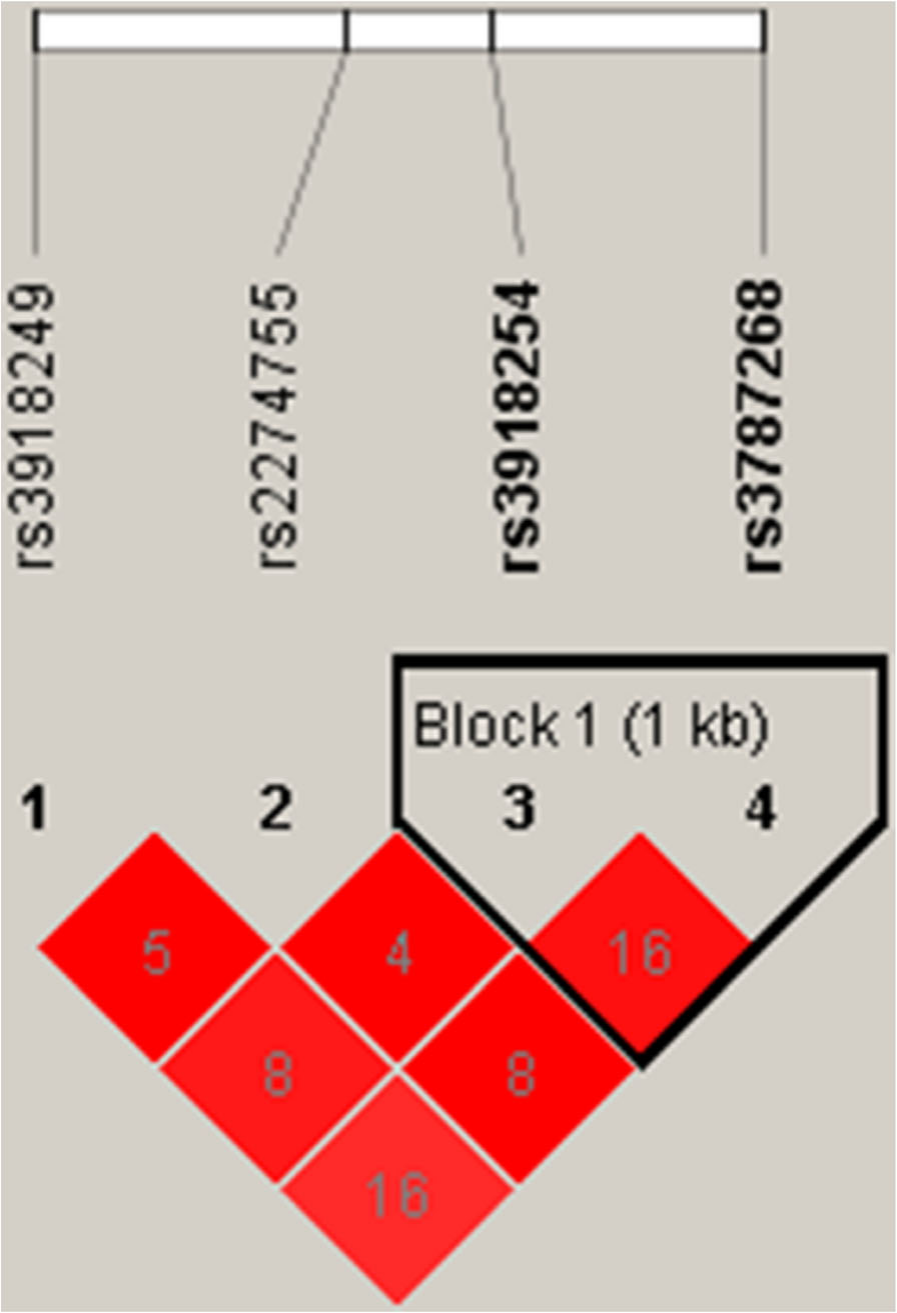
Haplotype block map for the four *MMP-9* single nucleotide polymorphisms explored in our study

We further evaluated the association between *MMP-9* haplotype and risk of developing IS. Figure 1 shows the LD of rs3918254 and rs3787268 in *MMP-9*. Haplotype “CG” was significantly associated with decreased risk of IS (OR = 0.71; 95% CI = 0.54–0.95; *p* = 0.019) (Table 4).

**Table 4:** MMP-9 haplotype frequencies and the association with the IS risk

Haplotype	rs3918254	rs3787268	Freq	OR(95%CI)	*p*
1	C	A	0.404	1.00	–
2	C	G	0.373	0.71 (0.54–0.95)	0.019^*^
3	T	G	0.221	0.82 (0.60–1.14)	0.240
rare	*	*	0.003	0.80 (0.05–13.09)	0.870

* *p* value ≤ 0.05 indicates statistical significance

*Freq* frequency, *ORs* odds ratios, *CI* confidence interval

We used HaploReg (version 4.1) to identify the rs3787268 tagged variants using the LD information from the 1000 Genomes Project (EUR) with r ^2^ ≥ 0.8, and we got 13 genetic variants tagged by rs3787268 variant with r ^2^ ≥ 0.8. These 13 genetic variants were located around the 18kb 5' of *MMP9*, *MMP9*, *RP11-465L10.7* and *SLC12A5*. The detailed information including the LD information about these variants was provided in Table 5.

**Table 5:** rs3787268 and variants with *r*^2^ ≥ 0.8

SNP	chr	pos (hg38)	LD (r^2^)	LD (D’)	Ref	Alt	Gene	Functional annotation
rs25610751	20	45,991,015	0.87	0.94	C	G	18 kb 5′ of *MMP9*	
rs6073980	20	45,991,065	0.86	0.94	G	A	18 kb 5′ of *MMP9*	
rs6073983	20	46,001,252	0.89	0.98	A	T	18 kb 5′ of *MMP9*	
rs3761157	20	46,006,183	98	0.99	C	T	18 kb 5′ of *MMP9*	
rs3787268	20	46,013,092	1	1	G	A	*MMP9*	intronic
rs3918262	20	46,015,131	1	1	A	G	*MMP9*	intronic
rs6073989	20	46,022,571	0.95	0.98	G	T	*RP11-465 L10.7*	intronic
rs16991010	20	46,023,675	0.95	0.98	A	C	*RP11-465 L10.7*	intronic
rs6130998	20	46,024,480	0.95	0.98	C	T	*RP11-465 L10.7*	intronic
rs6130999	20	46,026,853	0.95	0.98	G	A	*RP11-465 L10.7*	intronic
rs6073991	20	46,027,473	0.95	0.98	A	G	*RP11-465 L10.7*	intronic
rs13039389	20	46,031,067	0.91	0.97	G	C	*SLC12A5*	intronic
rs6131001	20	46,032,717	0.94	0.98	C	T	*SLC12A5*	intronic

*LD* linkage disequilibrium, *SNP* single nucleotide polymorphism, *Ref* reference allele, *Alt* altered allele

## Discussion

In this case-control study, we investigated four SNPs (rs3787268, rs3918249, rs2274755, rs3918254) of *MMP-9* to determine if they are significantly associated with the risk of IS in a southern Chinese Han population. Our results suggested SNP rs3787268 correlated with an increased risk of stroke; although, no significant relationship was found between IS and SNPs rs3918249, rs2274755, and rs3918254.

SNP rs378726 is located within *MMP-9* on chromosome 20q13.12 and may affect the development and progression of various diseases. For example, Ho et al. [20] found that rs378726 was associated with susceptibility to spontaneous deep cerebral hemorrhage in a Taiwanese population, while the minor allele of rs3787268 may exert a critical protective effect against diabetes [21]. In addition, rs3787268 has been used to predict the survival rate of breast cancer in a Chinese population [22]. However, the potential association between SNP rs378726 and IS has not been well studied. Currently, only the study of Zhong et al. [18] has suggested that rs3787268 may be a risk factors for IS in a Guangdong Chinese population. Similarly, we found that this polymorphism may promote the occurrence of IS in a southern Chinese Han population, although the effect of rs3787268 polymorphism on IS in other groups needs to be investigated.

The other loci we explored (rs3918249, rs2274755, rs3918254) also correlate with various diseases. For example, rs2274755 is associated with asthma [22] and steroid-induced osteonecrosis of the femoral head [23], while rs3918249 is related to childhood asthma and glaucoma. The rs3918254 locus may confer susceptibility to primary angle-closure glaucoma [24]. However, prior to our current study, their impact on the risk of IS had not been explored, although we found no significant relationship between them. For the first time, we found no significant relationship between them and the risk of IS in a southern Chinese Han population.

Our results suggest that certain polymorphisms in *MMP-9* can affect the risk of IS in a southern Chinese Han population, but further research is merited. First, because our sample size was relatively small, large-sample studies are needed to confirm these findings. In addition, we need to conduct functional studies to determine the relevant mechanism(s) of *MMP-9* polymorphisms and their effect on IS risk [25].

## Conclusion

In conclusion, we found that polymorphisms of *MMP-9* are significantly associated to the risk of IS in a southern Chinese Han population, providing foundational data for additional investigations of the relationship between *MMP-9* and IS risk in different populations. Our results also provide new insight for future explorations of IS pathogenesis. This may provide clues for the evaluation of individual susceptibility to IS and enable IS patients to receive early prevention and treatment, thus reducing the harm of IS.

## Abbreviations

CIs: 95% confidence intervals
IS: Ischemic stroke
*MMP-9*: Matrix metalloproteinase-9
OR: Odds ratio
SNPs: Single nucleotide polymorphisms
